# Type-A aortic dissection manifesting as acute inferior myocardial infarction: 2 case reports

**DOI:** 10.1097/MD.0000000000017662

**Published:** 2019-10-25

**Authors:** Wenjun Wang, Jiahong Wu, Xin Zhao, Beian You, Chuanbao Li

**Affiliations:** a Department of Emergency, Chest Pain Center; b Department of Radiology; c Department of Cardiac Surgery, Qilu Hospital of Shandong University; d The Key Laboratory of Cardiovascular Remodeling and Function Research, Chinese Ministry of Education and Chinese Ministry of Public Health, Qilu Hospital, Shandong University, Jinan, Shandong, China.

**Keywords:** acute myocardial infarction, aortic dissection, cardiac catheterization, computed tomography angiography

## Abstract

**Rationale:**

Acute Type-A aortic dissection (AD) is a challenging clinical emergency. Despite advances in diagnosis and surgical techniques, the high surgical mortality rate of the condition persists. As a result of similarities in clinical symptoms, AD can mimic acute myocardial infarction (AMI). In this paper, we report 2 cases of patients with acute AD manifesting as inferior AMI.

**Patient concerns:**

Two patients with undetected AD were misdiagnosed with AMI; in such patients, the administration of thrombolytic therapy has disastrous consequences.

**Diagnoses:**

The patients were initially diagnosed with AMI in the emergency room, and then diagnosed with AD during catheterization.

**Interventions:**

The patients were transferred to the cardiac catheterization laboratory for primary coronary angiography. The initial attempt to selectively engage the coronary ostium was unsuccessful. Subsequent computed tomography angiography (CTA) confirmed AD from the aortic root to the abdominal aorta and dissection violations of the coronary ostium. The patients underwent emergency aortic root replacement.

**Outcomes:**

One patient recovered and was discharged 2 weeks later. At a 1-year follow-up examination, CTA indicated that this patient had made a full recovery. The other patient died 6 days after surgery.

**Lessons:**

As a result of similarities in clinical symptoms, AD can mimic AMI. Rapid diagnosis and treatment of AD is crucial. Difficulty during catheter engagement should raise the suspicion of acute Type-A AD.

## 1 Introduction

Aortic dissection (AD) is a life-threatening illness that has a wide range of manifestations.^[1]^ As a result of similarities in clinical risk factors and presentations, AD can mimic acute myocardial infarction (AMI).^[2]^ Patients with AD with an ST-elevation on electrocardiography (ECG) suggestive of AMI are easily misdiagnosed. AMI and AD are both critical illnesses that require rapid diagnosis and treatment in the emergency department.^[3]^ However, the treatment of AD differs from that of AMI. If patients with AD are given thrombolysis, their mortality rate increases significantly as a consequence of further rupture and uncontrolled bleeding.^[4]^ Irrespective of misdiagnosis, the treatment of Stanford Type-A AD with coronary malperfusion due to extension of the dissection membrane into a coronary ostium is challenging for emergency physicians.^[5]^ Here, we report the cases of two patients with Type-A AD misdiagnosed as AMI.

## 2 Case presentation

The patients provided informed consent for the publication of their clinical and radiological data.

### 2.1 Case 1

A 60-year-old man presented to our emergency department with acute chest pain that had persisted for the preceding 2 hours. On admission, his body temperature was 36.5 °C; pulse 70 beats/min (bpm); respiratory rate 20 breaths/min; and blood pressure 112/65 mmHg. There was history of hypertension and diabetes in the past 3 years. No obvious positive signs were found on physical examination. ECG showed sinus rhythm with a 2-mm ST-segment elevation in the inferior limb leads. He was diagnosed with AMI and transferred to the cardiac catheterization laboratory for primary percutaneous coronary intervention (PCI).

The initial attempt to selectively engage the left coronary artery and right coronary artery (RCA) was unsuccessful. Contrast injection through the pigtail catheter showed that the aortic cavity was divided into two chambers by a false pipe. This suggested that the coronary ostium originated from the false lumen of a Type-A AD. Subsequent CTA confirmed final diagnosis of Type-A AD from the aortic root to the abdominal aorta.

The patient underwent emergency aortic root replacement and elective RCA–ascending aorta coronary artery bypass graft surgery. During cardiac surgery, AD from the aortic root to the ascending aorta and dissection violations of the RCA ostium were confirmed.

**Figure 1:**
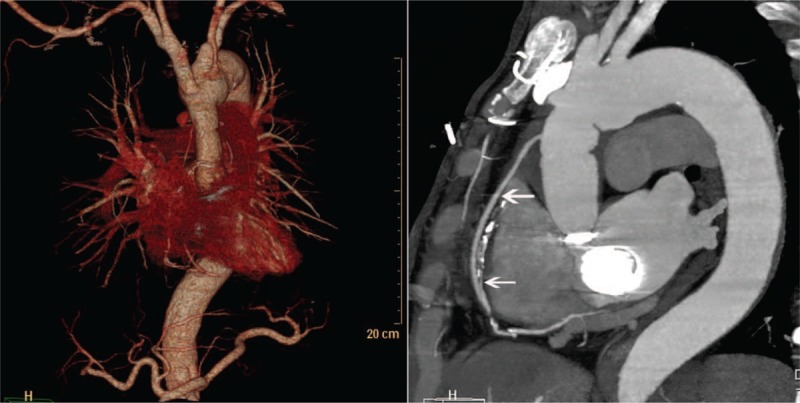
Computed tomography angiography showing that the ascending aorta is normal and that the saphenous vein graft to the right posterior descending artery remains patent (arrows).

The patient was discharged after 2 weeks. At a 1-year follow-up examination, computed tomography angiography (CTA) indicated that the patient had made a full recovery (Fig. 1).

### 2.2 Case 2

A 55-year-old woman was admitted to our emergency department with abdominal and back pain that had persisted for the preceding 7 hours. On admission, her body temperature was 36.0 °C; pulse 98 bpm; respiratory rate 23 breaths/min; and blood pressure 117/65 mmHg. No obvious positive signs were found on physical examination. Regarding her medical history, she had suffered from chronic hypertension for more than 10 years. ECG showed sinus rhythm with ST-segment elevation in the inferior limb leads. She was diagnosed with AMI and transferred to the cardiac catheterization laboratory for primary PCI.

**Figure 2:**
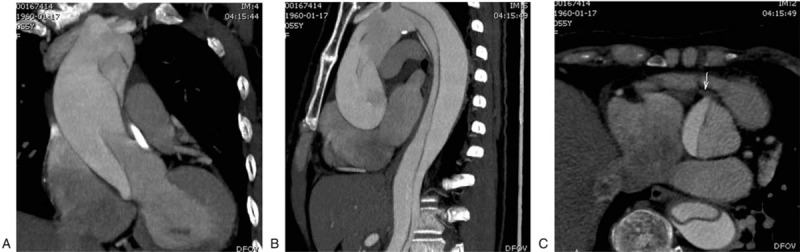
A and B: Multislice computed tomography reconstructions vividly showing aortic dissection from the aortic root to the abdominal aorta. C: Dissection violations of the right coronary artery ostium (arrows).

The initial attempt to selectively engage the coronary ostium was unsuccessful. Contrast injection through the pigtail catheter showed that the aortic cavity was divided into two chambers by a false pipe. Subsequent CTA confirmed Type-A AD from the aortic root to the abdominal aorta and dissection violations of the coronary ostium (Fig. 2).

The patient underwent emergency replacement of the ascending aorta, but died 6 days later.

## 3 Discussion

Acute Type-A AD is a challenging clinical emergency. Despite the advances in diagnosis and surgical techniques, the latest reports reveal a surgical mortality rate of more than 20%.^[6,7]^ As a result of similarities in clinical symptoms, AD can mimic AMI.^[2]^ Patients with undetected AD are often misdiagnosed with AMI. Patients with AMI and features suggestive of AD should be checked for the presence of AD to minimize the necessity of reperfusion therapy.

However, a short reperfusion time is associated with improved survival in patients with AMI. The 2017 European Society of Cardiology guidelines for ST-elevation myocardial infarction (STEMI) recommended a time from “STEMI diagnosis” to wire crossing of <90 minutes.^[8]^ Based on the concept that “time is myocardium”, AD is sometimes misdiagnosed as ST segment-elevation myocardial infarction and inappropriately treated with thrombolytic or PCI agents.^[9]^ Type-A AD can cause AMI when the dissecting membrane extends into a coronary ostium, and the expanding false lumen can compress the proximal coronary artery. The higher incidence of RCA involvement is attributable to the fact that dissection more commonly originates from the right anterior aspect of the ascending aorta above the right coronary sinus.^[10]^ Difficulty during catheter engagement should raise the suspicion of acute Type-A AD. D-dimer testing and transthoracic echocardiography (TTE) are helpful for the initial bedside screening of patients with chest pain, and CTA further aids the early recognition of AD.^[11]^

AD may mimic AMI due to similarities in clinical presentations and risk factors. To facilitate the differentiation of STEMI from AD, evaluation of the patient's complaints should focus on chest pain, associated symptoms, sex, hypertension, diabetes mellitus, and age-related differences in presentation. In 2010, the American Heart Association released guidelines for the diagnosis and management of patients with AD. In these guidelines, an aortic dissection detection risk score system was used as an initial clinical tool for the detection of AD.^[12]^

Our patients exhibited many indications of AD. In the emergency department, detailed characterization of chest pain may have provided clues to AD. In particular, the second patient complained of abdominal and back pain. Moreover, both patients had a history of uncontrolled hypertension. However, chest pain with ST elevation on ECG was suggestive of AMI. Fortunately, they were not given thrombolytic therapy and were transferred to the cardiac catheterization laboratory for coronary angiography. Unsuccessful attempts to selectively engage the coronary ostium aroused our suspicion. Then, subsequent CTA confirmed AD and the patients underwent emergency surgery. Unfortunately, the second patient died because her chest pain had persisted for more than 7 hours. Thus, rapid diagnosis and treatment of AD is crucial.

## 4 Conclusion

AD may indirectly cause AMI, and the possibility of AD should always be considered as a differential diagnosis in patients with AMI. TTE imaging, D-dimer testing, and AD detection risk score calculation may enable the early identification of AD. When there is a high index of suspicion, it is necessary to consider further imaging examinations.

## Acknowledgments

We would like to thank the native English-speaking scientists of Elixigen Company (Huntington Beach, CA) for editing our manuscript.

## Author contributions

**Conceptualization:** Beian You.

**Resources:** Wenjun Wang, Jiahong Wu, Beian You.

**Supervision:** Chuanbao Li.

**Visualization:** Xin Zhao.

**Writing – original draft:** Wenjun Wang, Chuanbao Li.

**Writing – review & editing:** Jiahong Wu, Xin Zhao.

## References

[1] Isselbacher EM Bonaca MP Di Eusanio M Recurrent aortic dissection: observations from the international registry of aortic dissection. Circulation 2016;134:1013 24. 27587434 10.1161/CIRCULATIONAHA.115.019359

[2] Koracevic GP Prehospital thrombolysis expansion may raise the rate of its inappropriate administration in ST-elevation acute myocardial infarction induced by aortic dissection. Am J Emerg Med 2013;31:628 9. 10.1016/j.ajem.2012.12.022 23380133

[3] Lentini S Perrotta S Aortic dissection with concomitant acute myocardial infarction: From diagnosis to management. J Emerg Trauma Shock 2011;4:273 8. 21769215 10.4103/0974-2700.82221 PMC3132368

[4] Núñez-Gil IJ Bautista D Cerrato E Incidence, management, and immediate and long-term outcomes after iatrogenic aortic dissection during diagnostic or interventional coronary procedures. Circulation 2015;131:2114 9. 25888682 10.1161/CIRCULATIONAHA.115.015334

[5] Hsieh TH Tsai LM Tsai MZ Characteristics of and atypical presentations in patients with acute aortic dissection -A single center experience. Acta Cardiol Sin 2011;27:238 43.

[6] Williams JB Peterson ED Zhao Y Contemporary results for proximal aortic replacement in North America. J Am Coll Cardiol 2012;60:1156 62. 22958956 10.1016/j.jacc.2012.06.023 PMC3699187

[7] Chikwe J Cavallaro P Itagaki S National outcomes in acute aortic dissection: influence of surgeon and institutional volume on operative mortality. Ann Thorac Surg 2013;95:1563 9. 23562465 10.1016/j.athoracsur.2013.02.039

[8] Ibanez B James S Agewall S 2017 ESC Guidelines for the management of acute myocardial infarction in patients presenting with ST-segment elevation: the Task Force for the management of acute myocardial infarctionin patients presenting with ST-segment elevation of the European Society of Cardiology (ESC). Eur Heart J 2018;39:119 77. 28886621 10.1093/eurheartj/ehx393

[9] Lee CH Lim J Type A aortic dissection: a hidden and lethal cause for failed thrombolytic treatment in acute myocardial infarction. Heart 2007;93:825. 17569805 10.1136/hrt.2006.100156 PMC1994447

[10] Sasaki S Watanabe H Shibayama K Three-dimensional transesophageal echocardiographic evaluation of coronary involvement in patients with acute type A aortic dissection. J Am Soc Echocardiogr 2013;26:837 45. 23759167 10.1016/j.echo.2013.05.001

[11] Luo JL Wu CK Lin YH Type A aortic dissection manifesting as acute myocardial infarction: still a lesson to learn. Acta Cardiol 2009;64:499 504. 19725443 10.2143/AC.64.4.2041615

[12] Rogers AM Hermann LK Booher AM Sensitivity of the aortic dissection detection risk score, a novel guideline-based tool for identification of acute aortic dissection at initial presentation: results from the International Registry of Acute Aortic Dissection. Circulation 2011;123:2213 8. 21555704 10.1161/CIRCULATIONAHA.110.988568

